# An Anti-MICA/B Antibody and IL-15 Rescue Altered NKG2D-Dependent NK Cell Responses in Hepatocellular Carcinoma

**DOI:** 10.3390/cancers12123583

**Published:** 2020-11-30

**Authors:** Stefania Mantovani, Stefania Varchetta, Dalila Mele, Matteo Donadon, Guido Torzilli, Cristiana Soldani, Barbara Franceschini, Camillo Porta, Silvia Chiellino, Paolo Pedrazzoli, Roberto Santambrogio, Matteo Barabino, Claudia Cigala, Gaetano Piccolo, Enrico Opocher, Marcello Maestri, Angelo Sangiovanni, Stefano Bernuzzi, Florence Lhospice, Manel Kraiem, Mario Umberto Mondelli, Barbara Oliviero

**Affiliations:** 1 Division of Infectious Diseases and Immunology, Fondazione IRCCS Policlinico San Matteo, 27100 Pavia, Italy; s.mantovani@smatteo.pv.it (S.M.); s.varchetta@smatteo.pv.it (S.V.); d.mele@smatteo.pv.it (D.M.); b.oliviero@smatteo.pv.it (B.O.); 2 Department of Hepatobiliary and General Surgery, Humanitas Clinical and Research Center, Humanitas University, 20089 Rozzano, Italy; matteo.donadon@hunimed.eu (M.D.); guido.torzilli@humanitas.it (G.T.); cristiana.soldani@humanitas.it (C.S.); barbara.franceschini@humanitas.it (B.F.); 3 Department of Medical Sciences and Human Oncology, “Aldo Moro” University of Bari and Policlinico Consorziale, 70124 Bari, Italy; camillo.porta@gmail.com; 4 Division of Medical Oncology, Fondazione IRCCS Policlinico San Matteo, 27100 Pavia, Italy; s.chiellino@smatteo.pv.it (S.C.); p.pedrazzoli@smatteo.pv.it (P.P.); 5 Division of General Surgery, ASST Fatebenefratelli—Sacco, 20131 Milan, Italy; rsantambrogio@mclink.it; 6 Division of Gastrointestinal Surgery, ASST Santi Paolo e Carlo, and State University of Milan, 20142 Milan, Italy; matteo.barabino@asst-santipaolocarlo.it (M.B.); claudia.cigala@asst-santipaolocarlo.it (C.C.); gpiccolo1983@gmail.com (G.P.); enrico.opocher@unimi.it (E.O.); 7 Division of General Surgery, Fondazione IRCCS Policlinico San Matteo, 27100 Pavia, Italy; mmaestri@smatteo.pv.it; 8 Division of Gastroenterology and Hepatology, CRC “A. M. and A. Migliavacca” Center for Liver Disease, Foundation IRCCS Ca’ Granda Ospedale Maggiore Policlinico, 20122 Milan, Italy; angelo.sangiovanni@policlinico.mi.it; 9 Immunohematology and Transfusion Service, Centre of Transplantation Immunology, Fondazione IRCCS Policlinico San Matteo, 27100 Pavia, Italy; s.bernuzzi@smatteo.pv.it; 10 Innate Pharma, 13009 Marseille, France; flhospice@emergencetx.com (F.L.); Manel.KRAIEM@innate-pharma.fr (M.K.); 11 Department of Internal Medicine and Therapeutics, University of Pavia, 27100 Pavia, Italy

**Keywords:** natural killer cells, HCC, sorafenib, immunotherapy, innate immunity

## Abstract

**Simple Summary:**

Primary liver cancer is one of the most common causes of cancer-related deaths worldwide. The present study shows that the cytokine IL-15 and a humanized antibody specific for a ligand recognized by a major activating receptor (NKG2D) expressed by a subset of lymphocytes named natural killer (NK) cells can restore dysfunctional NK cell responses in patients affected by the most common liver cancer hepatocellular carcinoma. This study emphasizes the importance of NK cells for immune responses to hepatocellular carcinoma and identifies the NKG2D activating receptor/ligand axis as a possible target for immunotherapeutic interventions. More importantly, building upon previously published studies, these findings provide a basis for the future treatment of tumors unresponsive to current therapies.

**Abstract:**

Natural killer (NK) cells play a pivotal role in cancer immune surveillance, and activating the receptor/ligand interaction may contribute to control the development and evolution of hepatocellular carcinoma (HCC). We investigated the role of the natural killer group 2 member D (NKG2D) activating receptor and its ligand, the major histocompatibility complex class I chain-related protein A and B (MICA/B) in patients with cirrhosis and HCC subjected to surgical resection, patients with cirrhosis and no HCC, and healthy donors (HD). The NKG2D-mediated function was determined in peripheral blood (PB), in tumor-infiltrating lymphocytes (NK-TIL), and in matched surrounding liver tissue (NK-LIL). A group of patients treated with sorafenib because of clinically advanced HCC was also studied. A humanized anti-MICA/B monoclonal antibody (mAb) was used in in vitro experiments to examine NK cell-mediated antibody-dependent cellular cytotoxicity. Serum concentrations of soluble MICA/B were evaluated by ELISA. IL-15 stimulation increased NKG2D-dependent activity which, however, remained dysfunctional in PB NK cells from HCC patients, in line with the reduced NKG2D expression on NK cells. NK-TIL showed a lower degranulation ability than NK-LIL, which was restored by IL-15 stimulation. Moreover, in vitro IL-15 stimulation enhanced degranulation and interferon-γ production by PB NK from patients at month one of treatment with sorafenib. Anti-MICA/B mAb associated with IL-15 was able to induce PB NK cytotoxicity for primary HCC cells in HD and patients with HCC, who also showed NK-TIL degranulation for autologous primary HCC cells. Our findings highlight the key role of the NKG2D-MICA/B axis in the regulation of NK cell responses in HCC and provide evidence in support of a potentially important role of anti-MICA/B mAb and IL-15 stimulation in HCC immunotherapy.

## 1. Introduction

Primary liver cancer is the 4th most common cause of cancer-related deaths worldwide [1]. Hepatocellular carcinoma (HCC) accounts for approximately 90% of primary liver cancers and often develops in a background of chronic viral hepatitis, alcoholic liver disease, or non-alcoholic steatohepatitis (NASH), after a multistep process requiring chronic necroinflammation and eventually leading to liver fibrosis and cirrhosis [2]. HCC treatment options have improved considerably over the last few years. Indeed, along with the most common first-line treatments, such as surgical resection, locoregional approaches (thermal ablation and transarterial chemoembolization), and liver transplantation, several drugs have been developed for the systemic treatment of patients with advanced disease. The first drug approved was the tyrosine kinase inhibitor sorafenib, which reduces tumor proliferation and angiogenesis by blocking the activities of vascular endothelial growth factor receptors (VEGFRs), platelet-derived growth factor receptors, and Raf family kinases, but extends the mean overall survival time of patients by only 2.8 months [3]. Moreover, because of its low tolerability, treatment discontinuation could occur in over 40% of patients [4,5,6]. With the advent of checkpoint inhibitors, new second-line treatment options have become available. In the HCC setting, treatments with the anti-PD1 monoclonal antibodies Nivolumab or Pembrolizumab were well tolerated and effective, resulting in encouraging response rates in patients enrolled in phase I and II trials [7]. Moreover, the use of immune-cells stimulated with cytokines could represent a new therapeutic option supported by findings in a mouse model [8,9]. Despite a promising role for immunotherapy in this clinical setting, there are currently very few studies addressing the immunopathogenesis of HCC, which may have important implications in the design of immunotherapeutic strategies. Several studies point to the importance of natural killer (NK) cells, and changes in NK cell frequency and phenotype have been described during HCC development in a transgenic mouse model of aggressive human liver cancer [10] as well as in human HCC [11]. Moreover, a positive correlation has been shown between the frequency of circulating and intrahepatic NK cells and survival in patients with HCC [12]. NK cell function is finely tuned by activating and inhibitory signals generated by the interaction of receptors with their ligands expressed on target cells [13]. The low affinity FcγRIIIa (CD16) is one of the most potent activating receptors and is associated with an immunoreceptor tyrosine-based activation motif (ITAM)-containing CD3 ζ−chain, allowing NK cells to recognize and kill antibody-coated target cells via antibody-dependent cellular cytotoxicity (ADCC) upon activation [14]. ADCC has been associated with better outcomes for some types of cancer [15,16]. Spontaneous NK cell lytic activity is mediated by several activating receptor-ligand axes, including the natural killer group 2, member D (NKG2D) activating receptor, and its ligands. NKG2D is a dimeric, type II membrane protein constitutively expressed by NK cells in humans, as well as by almost all CD8+ αβ T cells and γδ T cells [17]. NKG2D receptors recognize several molecules, such as MHC class I chain-related molecules (MIC)A/B and the cytomegalovirus UL-16 protein (ULBP1-6), the expression of which is induced on the cell surface as a result of cellular stress conditions, ranging from the activation of DNA-damage response pathways during tumorigenesis [18]. Therefore, NKG2D plays a pivotal role in immune surveillance and antitumor immune responses [19,20,21]. The expression of the NKG2D ligand MICA/B is dysregulated in various tumor cells, including HCC, which express MICA/B, their level of expression being correlated with the outcome of the disease. Interestingly, it has been shown that patients with reduced MICA expression in tumor specimens have a significantly shorter disease-free and overall survival expectancy than patients with preserved MICA expression [22,23]. It is also worth noting that modulation of MICA/B on HCC cells may represent a tumor escape mechanism. Indeed, shedding of NKG2D ligands due to metzincin-mediated cleavage [24], may yield soluble molecules that can bind to, and downregulate, NKG2D expression, impairing NKG2D-mediated immune responses [25]. Alternatively, downregulation of NKG2D on NK cells can be due to a transforming growth factor (TGF)β-mediated effect, the plasma levels of which were inversely correlated with NKG2D expression in patients with lung or colorectal cancer [26]. Although these data support a role for NK cells in HCC immune surveillance, the pathogenetic mechanisms leading to HCC development are poorly understood. Here we analyzed the characteristics of circulating and liver-infiltrating NK cells of patients with HCC who underwent tumor resection or medical treatment to identify druggable dysfunctional NK cell pathways.

## 2. Results

### 2.1. Defective Circulating NK Cell NKG2D-Mediated Function in HCC Patients Can Be Partially Restored by IL-15 Stimulation

**Figure 1:**
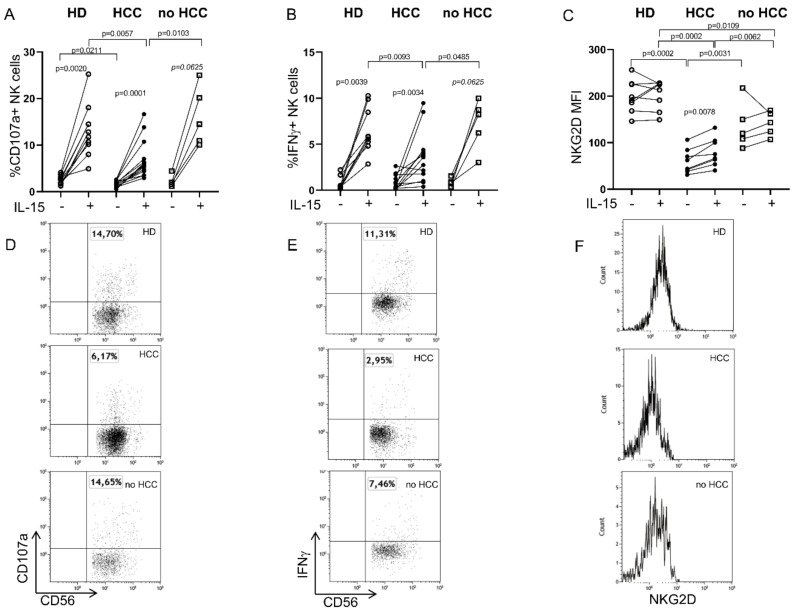
The impaired natural killer group 2, member D (NKG2D)-mediated function is partially restored by IL-15 stimulation in patients with hepatocellular carcinoma (HCC). Peripheral blood (PB) natural killer (NK) cell degranulation (% CD107a+ NK cells) (**A**) and IFNγ production (% IFNγ+ NK cells) (**B**) were measured in a redirected antibody-dependent cellular cytotoxicity (ADCC) assay (rADCC) by triggering the NKG2D receptor with an anti-NKG2D mAb—either unstimulated (-) or, after overnight stimulation, with IL-15 (+). HD, Healthy donors, *n* = 10 (**A**) and *n* = 9 (**B**); HCC, patients with HCC, *n* = 14 (**A**) and *n* = 12 (**B**); no HCC, cirrhotic patients without HCC, *n* = 5 (**A**,**B**). (**D**,**E**): Representative dot plots show NK cell function (degranulation and IFNγ secretion) after stimulation. NKG2D expression (Mean Fluorescence Intensity, MFI) (**C**) with or without IL-15 stimulation in HD (*n* = 8), HCC (*n* = 8) and no HCC (*n* = 5) subjects. Histograms show the MFI on unstimulated NK cells (**F**). The Mann–Whitney U test or Wilcoxon matched-pairs signed rank test were used to compare data.

Based on our previous findings [11], which highlighted alterations in the NKp30 receptor in patients with HCC and their role in anti-tumor response, we focused here on NKG2D, a major activating receptor. PB NK cell features were analyzed by flow cytometry in patients with HCC emerging in the context of cirrhosis (HCC), as well as in a small group of cirrhotic patients without HCC (no HCC) whose clinical characteristics are reported in Table S1, and in HD. Total PB NK and the CD56^dim^ and CD56^bright^ subsets, analyzed as described in Figure S1A, were equally distributed in all groups studied (Figure S1B). The PB NK function was then assessed by redirected antibody-dependent cell-mediated cytotoxicity (rADCC), by specifically triggering NKG2D with an anti-NKG2D mAb in the presence of P815 target cells bearing Ig Fc receptors, with or without prior stimulation with IL-15, an emerging tool to harness NK cell-mediated anti-cancer immunotherapy [27,28]. As expected, IL-15 stimulation significantly increased NK cell cytotoxicity and cytokine production in both HD and patients, but it was unable to fully restore either the PB NK cell function (Figure 1A,B) or NKG2D expression (Figure 1C), which remained lower than in HD and in no HCC patients.

**Figure 2:**
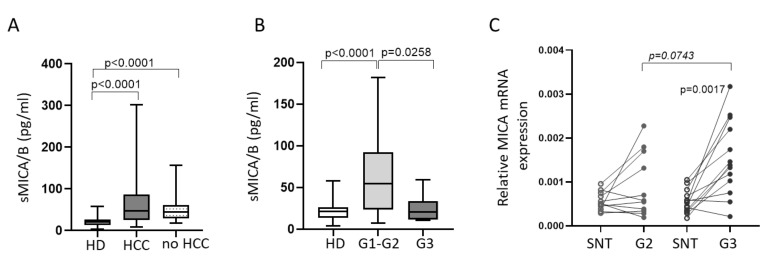
sMICA/B concentrations are increased in patients with HCC. (**A**) Soluble MICA/B (sMICA/B) was tested in sera of 45 HD, 73 HCC, and 19 no HCC subjects. (**B**) sMICA/B concentrations in HCC patients stratified according to the grades of tumor differentiation G1, G2, and G3 (well, moderately, and poorly differentiated, respectively). G1: *n* = 24; G2: *n* = 18; G3: *n* = 10. (**C**) mRNA levels in G2 (*n* = 11) and G3 (*n* = 13) tumors and in their matched surrounding non-tumor (SNT) specimens. Middle bars represent median values, box plots are 25% and 75% percentiles, whiskers are minimum and maximum values. The Mann–Whitney U test or Wilcoxon matched-pairs signed rank test were used to compare data.

Since the release of the NKG2D ligand MICA/B could represent a tumor evasion mechanism resulting in the downregulation of NKG2D with an ensuing impaired NK cell function [25,29], we tested the hypothesis that decreased NKG2D expression on PB NK in patients could be associated with an increased soluble form of MICA/B (sMICA/B). To this aim, we tested sMICA/B in sera from HD and patients. As shown in Figure 2A, both HCC and no HCC patients showed higher sMICA/B levels compared with HD. There was no correlation between sMICA/B levels and the parameters of liver disease, including aspartate aminotransferase to platelet ratio index (APRI) and fibrosis-4 (FIB-4) score; however, patients with poorly differentiated HCC (G3) had lower concentrations of sMICA/B compared with those bearing well (G1) or moderately (G2) differentiated tumors (Figure 1B). To understand if the decrease in serum sMICA/B concentrations in patients with G3 HCC was due to a reduction in gene transcription, we measured MICA mRNA levels in G2 and G3 tumor tissue and in their matched surrounding non-tumor (SNT) specimens. Only poorly differentiated tumor tissue showed significantly increased MICA transcription compared to matched SNT tissue. G3 tissue also showed a trend toward increasing MICA mRNA levels compared to G2 tissue, suggesting that other post-transcriptional mechanisms are involved in the regulation of MICA expression in G3 specimens (Figure 1C).

### 2.2. IL-15 Restores NKG2D-Mediated Function in Tumor-Infiltrating NK Cells

To determine the characteristics of NK cells in the liver compartment and to assess their possible role in tumor surveillance, tumor and matched adjacent infiltrating lymphocytes (TIL and LIL, respectively) from 26 HCC patients subjected to tumor resection were analyzed. As shown in Figure S2A, the frequency of NK cells was decreased in the tumor compartment, in keeping with our previously published findings [11], with a lower proportion of CD56^dim^ and a higher proportion of CD56^bright^ NK cells (Figure S2B,C, respectively).

**Figure 3:**
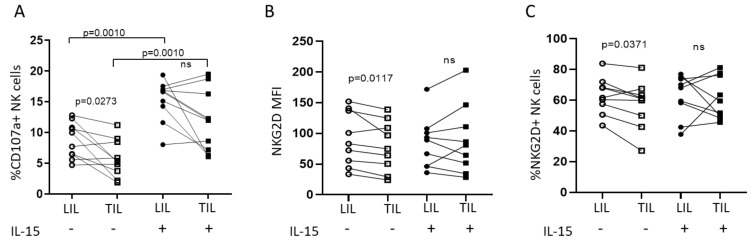
IL-15 stimulation induced NKG2D-mediated degranulation in intrahepatic NK cells. Cytotoxic degranulation (% of CD107a+ NK cells) (**A**) was examined in a rADCC assay without (-) and after IL-15 stimulation (+) of liver-infiltrating lymphocytes (LIL) (*n* = 10) and matched tumor-infiltrating lymphocytes (TIL) (*n* = 10). NKG2D expression (Mean Fluorescence Intensity, MFI) (**B**) and NKG2D+ NK cell frequency (% NKG2D+ NK cells) (**C**) were measured in the same conditions in NK-LIL (*n* = 9) and matched NK-TIL (*n* = 9). The Mann–Whitney U test or Wilcoxon matched-pairs signed rank test were used to compare data; ns = not significant.

A functional analysis of intrahepatic NK cells showed that NKG2D-mediated cytotoxicity by NK-TIL was impaired compared with NK-LIL (Figure 3A), in agreement with the lower NKG2D expression and the frequency of NKG2D expressing NK cells (Figure 3B,C, respectively). The stimulation with IL-15 significantly increased the NK-LIL and NK-TIL NKG2D-mediated degranulation and was able to restore NK-TIL functionality, which became comparable to that of stimulated NK-LIL (Figure 3A and Figure S3A), in line with the NKG2D expression and frequency (Figure 3B,C and Figure S3B,C). Interestingly, IL-15 stimulation resulted in a decreased proportion of CD56^dim^ and a correspondingly increased proportion of CD56^bright^ NK cells in both LIL and TIL (Figure S2D,E), suggesting that the restoration of NK-TIL activity did not depend on the frequency of the cytotoxic NK cell subset but, rather, on the ability of IL-15 to restore NKG2D expression. Moreover, there was a correlation between NKG2D expression and degranulation in TIL. Notably, a lower NKG2D expression correlated with a lower CD107 expression, both in unstimulated and IL15-stimulated TIL-derived NK cells (Figure S4).

### 2.3. In Vitro IL-15 Stimulation Increases NK Cell Responses in Patients Treated with Sorafenib

**Figure 4:**
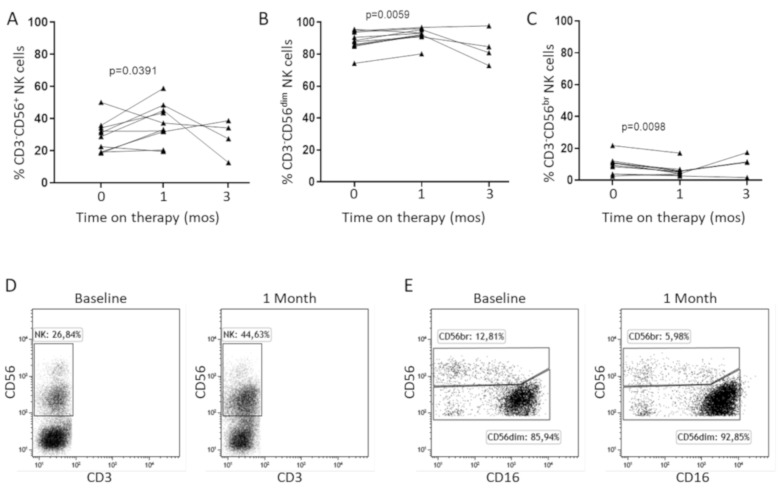
NK cell subset distribution in HCC patients treated with sorafenib. Frequencies of total (**A**), CD56^dim^ (**B**), and CD56^bright^ (**C**) NK cells in HCC patients before (*n* = 10) and after 1 (*n* = 10) and 3 (*n* = 4) months of sorafenib treatment. The Wilcoxon matched-pairs signed rank test was used to analyze data. Panel (**D**) and Panel (**E**) show representative dot plots of total CD56^dim^ or CD56^bright^ NK cells at baseline and 1 month on therapy.

To investigate the possible role of NKG2D in the response to HCC treatment, we studied patients at baseline and at two time points, 1 month and 3 months, on standard 1st line therapy with sorafenib. The analysis of PB NK distribution revealed a significant increase of total NK cells after 1 month on treatment (Figure 4A,D), consisting predominantly of CD56^dim^ NK cells (Figure 4B,C,E).

**Figure 5:**
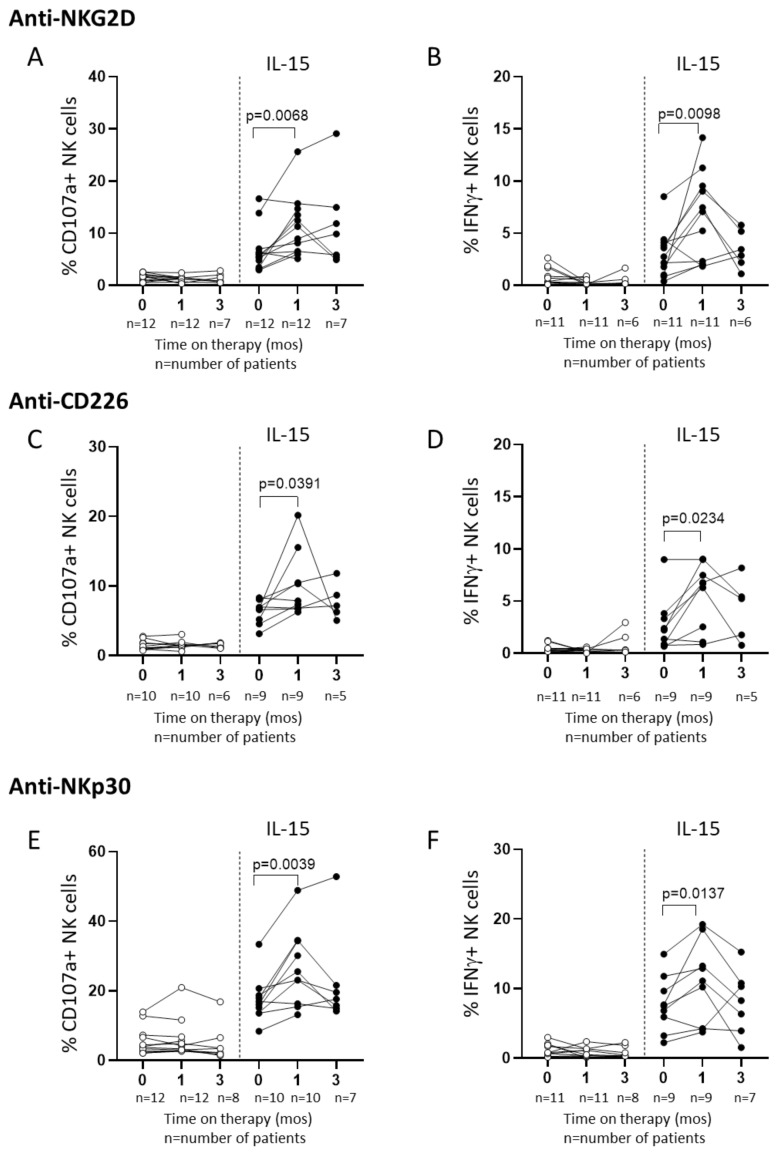
IL-15 stimulation restores the NK cell function in patients with HCC treated with sorafenib. NK cell degranulation and IFNγ production were evaluated before and after PBMC stimulation with IL-15 in rADCC assays by triggering NKG2D (**A**,**B**), CD226 (**C**,**D**), and NKp30 (**E**,**F**) receptors. The Wilcoxon matched-pairs signed rank test was used to analyze data.

We next tested the NK cell activity in rADCC experiments and found that NK degranulation and IFNγ production were comparable with baseline values during sorafenib therapy (Figure 5A,B, respectively), in line with NKG2D expression and the frequency of NKG2D+ NK cells (Figure S5A,B). The stimulation of NK cells with IL-15 in sorafenib-treated patients produced a significant increase in NKG2D-mediated CD107a expression and IFNγ production after 1 month on therapy, which, however, was not sustained over time (Figure 5A,B, IL-15 panels). Surprisingly, NKG2D expression did not change following IL-15 stimulation (Figure S5A,B, IL-15 panels), suggesting that the IL-15 effect on NK activity was independent of the NK cell receptor. To explore this hypothesis, we tested the NK cell function mediated by other activating receptors, CD226 and NKp30, in rADCC assays. Interestingly, CD226 (Figure 5C,D) or NKp30 (Figure 5E,F) ligation showed identical short-term responses observed with NKG2D stimulation both in terms of degranulation and IFNγ production. Indeed, sorafenib alone induced no changes in the NK cell function 1 month after treatment commencement, whereas in vitro IL-15 stimulation significantly boosted NK cell responses. Moreover, the NK cell enhanced function was independent on CD226 and NKp30 expression, which was not modified after IL-15 stimulation (Figure S5C–F).

### 2.4. A Humanized Anti-MICA/B mAb Increases the Cytotoxic Potential of HCC-Infiltrating NK Cells

Based on previous findings which showed that a humanized anti-MICA/B mAb could enhance PB NK cell activity [30], we chose to use this mAb in association with IL-15 stimulation to maximize performance in in vitro cytotoxic experiments.

**Figure 6:**
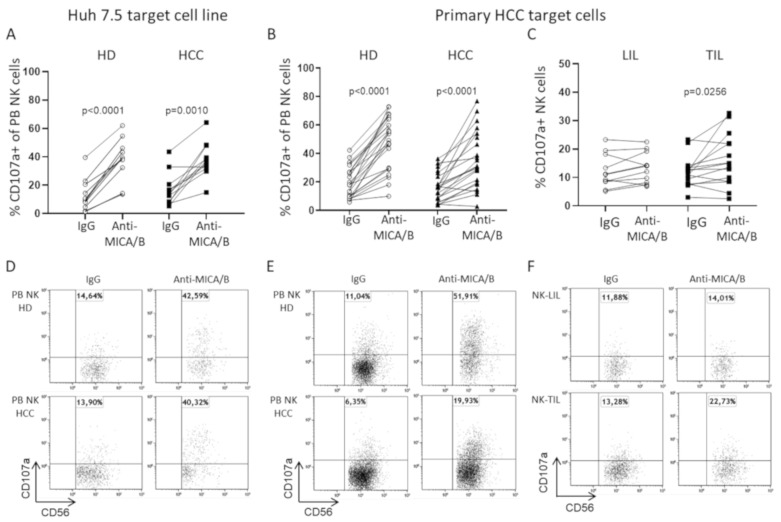
Effect of anti-MICA/B on NK-cell cytotoxicity for Huh 7.5 or primary HCC target cells in HD and HCC patients. (**A**): HD (*n* = 19) and HCC (*n* = 12) PB NK degranulation toward Huh 7.5 target cells after stimulation with IL-15 in the presence of control isotype (IgG) or anti-MICA/B mAb (anti-MICA/B). (**B**): CD107a expression evaluated on IL-15 stimulated PB NK of HD (*n* = 24) and HCC (*n* = 22) toward allogeneic or autologous primary HCC cells obtained from resected tumors, respectively. (**C**): Degranulation of IL-15 stimulated NK-LIL (*n* = 12) and matched NK-TIL (*n* = 15) in the presence of IgG control isotype or anti-MICA/B toward autologous primary HCC cells as targets. (**D**,**E**,**F**): Representative dot plots showing degranulation by IL-15 stimulated PB NK of HD and HCC or NK-LIL and NK-TIL toward target cells. The Mann–Whitney U test or the Wilcoxon matched-pairs signed rank test were used to analyze data.

In a first set of experiments, the MICA/B-expressing Huh 7.5 hepatocellular carcinoma cell line was used as target. The addition of anti-MICA/B mAb and IL-15 to the system determined a significant increase in PB NK degranulation both in HD and in HCC patients compared with the control isotype (IgG) (Figure 6A,D). Interestingly, anti-MICA/B mAb-mediated NK cell degranulation in patients with HCC was comparable with HD (Figure 6A,D). MICA/B+ primary HCC cells isolated from tumor resections were used as target cells in a second set of experiments in which the anti-MICA/B mAb significantly increased PB NK degranulation activity for both allogeneic HCC primary cells in HD and autologous cells in the case of HCC patients (Figure 6B,E). The liver-infiltrating NK cytotoxic potential for autologous primary HCC target cells was also examined in IL-15 stimulated NK-LIL and NK-TIL in the presence of anti-MICA/B mAb or its control isotype. Interestingly, anti-MICA/B mAb significantly increased the degranulation in NK-TIL, but not in NK-LIL cells (Figure 6C,F). Importantly, as shown in Figure S7, there was a statistically significant correlation between the expression of CD16 (FcγRIIIa) on NK cells and the difference between degranulation with an anti-MICA/B mAb and IgG isotype control (∆ Anti-MICA/B—IgG). These data provide an explanation for the relatively few points contributing to the differences in CD107a expression observed following the addition of anti-MICA/B to the system, since the efficiency of ADCC was clearly dependent upon the expression of CD16.

ADCC was also determined by measuring 7-aminoactinomycin D (7-AAD) expression, which may more faithfully reflect target cell killing. Only HD PB NK could significantly kill primary HCC cells in the presence of anti-MICA/B mAb, whereas no significant differences in 7-AAD expression were observed in primary HCC cells, in the presence of autologous HCC PB NK, NK-LIL, or NK-TIL (Figure S6A–C). To assess whether NK cytotoxic function could depend upon MICA/B expression, we checked the expression of MICA/B on primary HCC cell lines, and a relationship was found with target cell killing using PBMC as effectors, but not when using LIL or TIL, suggesting that other factors, such as the inhibitory liver microenvironment, may negatively influence NK cell function in this setting (Figure S6D). Interestingly, patient PB NK cells showing the highest cytotoxicity values with anti-MICA/B in the 7-AAD target labeling assay displayed high CD16 expression (Figure S8).

**Figure 7:**
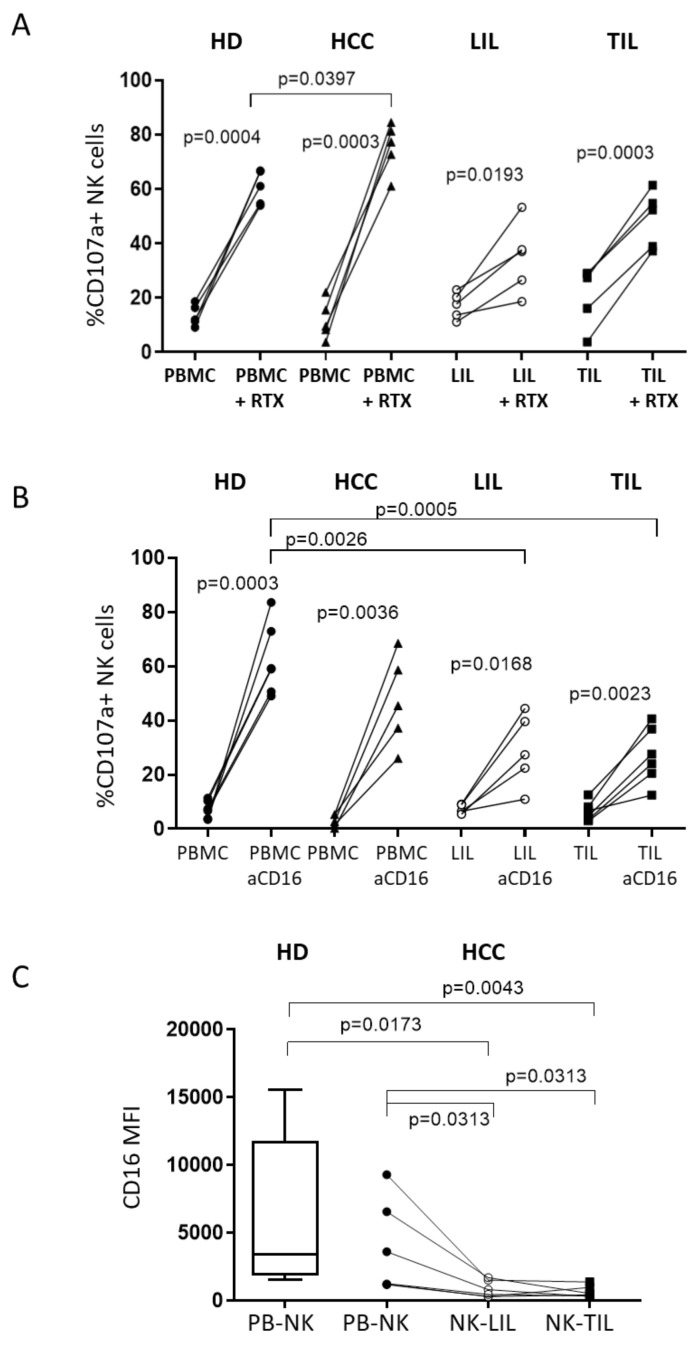
CD16-mediated NK activity. (**A**): NK cell cytotoxicity of HD (*n* = 6) and HCC patients (peripheral and liver compartments, *n* = 5) for Daudi cells after stimulation with IL-15 before and after the addition of anti-CD20 mAb (+RTX). (**B**): CD107a expression in NK cells from HD (*n* = 5) and HCC patients (peripheral and liver compartments, *n* = 5) after stimulation with IL-15, with or without coating with anti-CD16 (aCD16) mAb. (**C**): Expression of CD16 on patients PB-NK, NK-LIL, and NK-TIL.

To investigate whether ADCC activity was influenced by an altered anti-MICA/B mAb-CD16 interaction, we tested the ability of an anti-CD20 mAb (rituximab, RTX) to mediate ADCC for Daudi target cells derived from a human B-cell lymphoma. PB NK cells from HD and peripheral and matched intrahepatic NK cells from HCC patients were used in the experiments. PB NK cells from both HD and patients with HCC, with their matched NK-LIL and NK-TIL, were able to degranulate significantly more efficiently in the presence of RTX compared with Daudi target cells alone, although both NK-LIL and NK-TIL showed a statistically non-significant reduced degranulation compared with PB NK (Figure 7A). This is in line with the CD16-mediated response assessed after a direct CD16 ligation with an anti-CD16 mAb (Figure 7B) and with their lower CD16 expression, as shown in Figure 7C.

## 3. Discussion

Innate immune responses are key in the pathogenesis of several human cancers, including hepatocellular carcinoma (HCC) [10,11,12,13]. Therefore, NK cells represent a promising tool in anti-cancer immunotherapeutic strategies, including allogeneic NK infusion in several limpho- and myeloid-proliferative disorders (reviewed in [31]), and the most recent innovative approaches based on the use of NK receptor inhibitors [32] and therapeutic chimeric antigen receptor (CAR)-NK [33,34]. Here we built upon our previous study [11], which highlighted alterations in NKp30-B7H6 axis in patients with HCC, and focused on the major NKG2D-MICA/B receptor-ligand axis, that is known to play a principal role in anti-tumor surveillance.

Previous studies found that the down-modulation of MICA/B on tumor cells could affect the outcome of the disease. Indeed, patients with reduced MICA expression on HCC specimens had a significantly shorter disease-free and overall survival expectancy than patients with a preserved MICA expression [22,23]. The decreased MICA/B exposure on tissue could be due to the metzincin-mediated cleavage and shedding of sMICA/B in serum [24]. Notably, previous evidence suggests that the binding of sMICA/B to the NKG2D receptor determined its downregulation on immune effector cells, causing impaired NKG2D-mediated immune responses [25]. In keeping with such findings, we showed that increased levels of sMICA/B in the serum of patients with cirrhosis with or without HCC was associated with a lower expression of NKG2D on PB NK. Intriguingly, subjects bearing a poorly differentiated tumor (G3) had a decreased sMICA/B concentration. This finding could be due to post-transcriptional regulation, such as proteasomal degradation, as described by Fang and colleagues [35], since MICA mRNA levels in poorly differentiated (G3) tumors were comparable with moderately differentiated (G2) tumors.

Intrahepatic tumor-infiltrating NK cells featured a hypofunctional NKG2D, consistent with their lower NKG2D expression. This latter finding is akin to data from Easom and colleagues [36], who demonstrated that the reduction of NKG2D on intrahepatic NK cells after exposure to a hepatoma cell line was due to the internalization of the receptor. We investigated the possibility of reverting the NK impaired functionality in patients with HCC by cytokine stimulation. The choice of IL-15 was based upon encouraging results obtained in mouse models [8,9,27] and on its use in several immunotherapeutic studies [28,37,38,39]. To this end, it is of interest that an NK cell line transfected with IL-15 signals in an autocrine fashion to control HCC in mouse models [40]. Unfortunately, IL-15 stimulation failed to rescue NKG2D-mediated activities and NKG2D expression in the circulating NK cell compartment in patients with HCC. However, note that IL-15 was able to restore the NK-TIL function, which is consistent with the improved receptor expression. Based on these findings, it is possible that a different NKG2D expression could influence the NK function, depending on the compartment analyzed. Therefore, IL-15 could interfere with tissue-dependent NKG2D regulation via a NKG2D internalization blockade following the interaction with ligands on target cells [36], which would explain why we did not observe the same degree of recovery on PB NK. Interestingly, the effect of IL-15 on PB NK was observed only when associated with short-term (1 month) sorafenib treatment, at a time when the degranulation and IFNγ production from stimulated NK cells significantly improved over the baseline. Importantly, this was also observed for several other receptor-mediated activities, including CD226 and NKp30, suggesting that the combined effect of sorafenib and IL-15 was not receptor-specific. No correlation was found between the NK cell function and the clinical response to sorafenib. With respect to this point, it is well known that the beneficial effects of sorafenib are limited in time and that resistance to anti-VEGF therapy often occurs due to escape mechanisms of the angiogenic process through the activation of signaling pathways other than the VEGF pathway. Indeed, this agent can inhibit up to 40 kinases, including mainly angiogenic RTKs (including VEGF receptors and PDGF receptor-β) and drivers of cell proliferation (such as RAF1, BRAF, and KIT) (reviewed in [41]).

Over the past few years, mAbs targeting T-cell checkpoint molecules have been developed, as (reviewed in [7]). These therapeutics have more recently been enriched by mAbs specific for NK checkpoint inhibitors that are currently being evaluated in phase I/II clinical trials for treatment of solid tumors and hematological malignancies [31]. Moreover, recent exciting results were obtained from Gauthier et al. [42], who tested the new generation of trifunctional NK cell engagers, which are multifunctional antibodies targeting NKp46 and CD16 activating receptors and tumor antigens. In line with these novel approaches, part of our study focused on CD16-mediated NK activity. Our results indicated that the CD16-mediated response, measured as degranulation after direct CD16 stimulation by coating with an anti-CD16 antibody, was impaired in NK-LIL and matched NK-TIL, which correlated with a lower CD16 expression. These findings were confirmed when the cytotoxic activity was examined using CD20-expressing Daudi target cells and an anti-CD20 antibody, confirming the trend toward reduced ADCC function mediated by NK derived from the liver compartment and corroborating the interpretation that CD16 expression is critical for optimal ADCC. In order to optimize ADCC and the NKG2D-MICA/B axis, we used a humanized anti-MICA/B mAb, which could on one side stimulate the NK CD16-mediated activity and on the other intercept the binding of NKG2D to the sMICA/B cell-expressed ligand, thus disrupting the interaction between MICA/B and NKG2D, with the ensuing impaired immunosurveillance [43,44]. Our findings showed that anti-MICA/B mAb significantly increased NK-TIL degranulation in the presence of primary human HCC cells. The data were corroborated by evidence that NK cells from HCC patients and HD displayed efficient cytotoxic potential when challenged with standard and primary HCC cell lines.

In conclusion, we have shown that in patients with HCC, the altered NK function caused by the impaired NKG2D-MICA/B interaction, could be restored by IL-15 stimulation associated with a humanized anti-MICA/B-specific mAb that efficiently mediates ADCC in vitro. Collectively, our findings provide the basis for potential NK cell-based immunotherapeutic interventions in the setting of HCC.

## 4. Materials and Methods

### 4.1. Patients and Biological Material

Paired peripheral blood mononuclear cells (PBMC) and surgically resected HCC specimens, along with matched non-neoplastic surrounding tissue, were obtained from patients admitted to Fondazione IRCCS Policlinico San Matteo, Pavia, IRCCS Humanitas Research Hospital, and ASST Santi Paolo e Carlo Hospital, Milan, Italy. Main patient characteristics are listed in Table S1. PBMC from patients treated with sorafenib were obtained from Fondazione IRCCS Policlinico San Matteo. A written informed consent was obtained from each individual. The study protocol is compliant with the ethical guidelines of the 1975 Declaration of Helsinki and was approved by our institutional ethical committee (protocol numbers: 20160004446, 20150000576, 201430031379).

### 4.2. RNA Extraction and qPCR

Tissue RNA extraction and cDNA synthesis were performed as previously described [8]. The SsoAdvanced Universal SYBR Green Supermix (BioRad, Hercules, CA, USA) was used. Each sample was amplified in triplicate, and the qPCR data were analyzed using the 2−ΔCt method. MICA mRNA expression was normalized to the glyceraldehyde 3-phosphate dehydrogenase gene. The following primers were used: GAPDH forward 5′-CGGATTTGGTCGTATTGG-3′ and reverse 5′-GGTGGAATCATATTGGAACA-3′; MICA forward 5′-TCCTGCTTCTGGCTGGCAT-3′ and reverse 5′-GACAGCACCGTGAGGTTA-3′ (Primm, Milan, Italy).

### 4.3. ELISA

Serum levels of soluble MICA/B (sMICA/B) were measured by a Human ELISA kit (Abcam, Cambridge, UK) in patients and healthy donors (HD), according to the manufacturer’s instructions.

### 4.4. Phenotypic and Functional Analysis

The isolation of PBMC and tissue-infiltrating lymphocytes was performed as described in the Supplementary Materials. A flow cytometry analysis of ex-vivo isolated PBMC, non-tumor liver-infiltrating lymphocytes (LIL), and tumor-infiltrating lymphocytes (TIL) was performed using a 9-color CyAn (Beckman Coulter, Brea, CA, USA) and a 12-color FACSCelesta (BD Biosciences, San Diego, CA, USA) instruments. The mouse anti-human monoclonal fluorescent antibodies (mAbs) used are listed in Table S2. A LIVE/DEAD^®^ Fixable Near-IR Dead Cell Stain Kit (Thermo Fisher Scientific, Waltham, MA, USA) was used to determine the cell viability. Briefly, 2 × 10^5^ PBMC, LIL or TIL, were stained with mAbs for 30 min at 4 °C, washed, immediately fixed in CellFix solution (BD Biosciences), and analyzed.

Details of cell lines and primary HCC cell cultures, as well as PBMC, LIL, and TIL staining, are reported in the Supplementary Materials. Briefly, for the redirecting assay, also named reverse antibody-dependent cellular cytotoxicity (rADCC) assay, unstimulated or IL-15 stimulated PBMC, TIL and LIL, were incubated with FcγR+ P815 murine target cells in the presence of anti-NKG2D, -CD226, or -NKp30 specific mAbs. In the ADCC assays, the effect of the control isotype (IgG) or humanized anti-MICA/B mAb (kindly provided by Innate Pharma, Marseille, France) was tested on Huh 7.5 or primary HCC targets cells. The CD16-mediated NK activity was also evaluated after direct CD16 stimulation or toward the Daudi cell line in the presence of rituximab.

### 4.5. Statistical Analysis

The statistical analysis was performed using the GraphPad Prism 6 software (GraphPad, La Jolla, CA, USA). Data distributions among groups were checked for normality (D’Agostino & Pearson test) prior to any analysis. Since the distribution was consistently not normal, we have used non-parametric tests for all our analyses, i.e., Wilcoxon matched-pairs signed rank test for paired data, the Mann–Whitney U test for unpaired data, and the Spearman correlation test.

A *p* value ≤ 0.05 was deemed statistically significant.

## 5. Conclusions

The NKG2D-mediated NK cell function was altered in hepatocellular carcinoma, because of inappropriately reduced NKG2D expression. IL-15 stimulation in combination with a humanized anti-MICA/B-specific mAb could restore NK cell activity through the reconstitution of efficient ADCC in vitro. Collectively, our findings provide the basis for potential NK cell-based immunotherapeutic interventions in the setting of HCC.

## Supplementary Materials

The following are available online at https://www.mdpi.com/2072-6694/12/12/3583/s1. Table S1: Clinical characteristics of patients, Table S2: Details of monoclonal antibodies used in the experiments, Materials and Methods, Figure S1: Circulating NK cell subset distribution in HD, HCC, and no HCC patients, Figure S2: NK cell subset distribution in LIL and TIL, Figure S3: NKG2D-mediated degranulation and NKG2D expression in NK-LIL and NK-TIL, Figure S4: Correlation between NKG2D expression and NK-TIL cell degranulation, Figure S5: Expression of activating receptors on NK cells of HCC patients treated with sorafenib, Figure S6: ADCC mediated by anti-MICA/B mAb, Figure S7: Correlation between CD16 expression and NK-TIL degranulation, Figure S8: CD16 expression on PB NK and their killing activity.
