# Supplementary material for: An Anti-MICA/B Antibody and IL-15 Rescue Altered NKG2D-Dependent NK Cell Responses in Hepatocellular Carcinoma
Source: Cancers (Basel). 2020 Nov 30;12(12):3583. doi: 10.3390/cancers12123583 (PMC7761065; doi:10.3390/cancers12123583)
Supplement: Supplementary file 1 [file cancers-12-03583-s001.pdf]

cancers

Supplementary materials: An Anti-MICA/B Antibody and IL-15 Rescue Altered NKG2D-Dependent NK Cell Responses in Hepatocellular Carcinoma

Stefania Mantovani, Stefania Varchetta, Dalila Mele, Matteo Donadon, Guido Torzilli, Cristiana Soldani, Barbara Franceschini, Camillo Porta, Silvia Chiellino, Paolo Pedrazzoli, Roberto Santambrogio, Matteo Barabino, Claudia Cigala, Gaetano Piccolo, Enrico Opocher, Marcello Maestri, Angelo Sangiovanni, Stefano Bernuzzi, Florence Lhospice, Manel Kraiem, Mario Umberto Mondelli and Barbara Oliviero

Table S1. Clinical characteristics of patients and controls.

	Cirrhosis HCC	%	Cirrhotic non-HCC	%
Number of subjects	122		19	
Male/Female	97/25	84/23	10/9	53/47
Median age (years) - range	70 (36-85)	-	68 (47-77)	-
ALT (mU/ml) median, range	51 (14-290)	-	65 (12-254)	-
AST (mU/ml), median, range	61 (16-326)	-	73 (15-187)	-
APRI, median, range	1.03 (0.18-7.97)	-	1.76 (0.16-3.21)	-
FIB-4, median, range	3.48 (0.84-21.15)	-	5.35 (1.35-12.41)	-
BCLC score				
A	61	50	na	-
B	31	25.4	na	-
C	28	22.9	na	-
D	2	1.64	na	-
CTP score				
A	103	84.4	18	94.7
B	16	13.1	1	5.3
C	3	2.5	0	0
MELD score				
≤9	83	68	14	73.7
10-19	38	31.2	5	26.3
≥20	1	0.8	0	0
HCC size** (mm), median, range	46 (18-172)	-	na	-
Etiology				
HBV	16	13.1	0	0
HCV	84	68.9	19	100
HBV + HCV	2	1.6	0	0
NASH	7	5.7	0	0
NASH + Ethanol	1	0.8	0	0
HCV + Ethanol	2	1.6	0	0
Ethanol	1	0.8	0	0
Unknown	9	7.4	0	0

*na: not applicable.
**Maximum nodule width.

ALT, alanine aminotransferase; AST, aspartate aminotransferase; APRI, aspartate aminotransferase to platelet ratio index; FIB-4, fibrosis-4; BCLC, Barcelona Clinic Liver Cancer; CTP, Child-Turcotte-Pugh; MELD, Model for End Stage Liver Disease; HBV, hepatitis B virus; HCV, hepatitis C virus; NASH, non-alcoholic steatohepatitis.

Table S2. Details of monoclonal antibodies used in the experiments.

Antigen	Manufacturer	Catalog Number
Phenotype		
CD3 FITC	BD Biosciences, San Diego, CA, USA	555332
CD3 BV421™	BD Biosciences, San Diego, CA, USA	562426
CD16 PE	BD Biosciences, San Diego, CA, USA	555407
CD45 BV786™	BD Biosciences, San Diego, CA, USA	563716
NKG2D APC	BD Biosciences, San Diego, CA, USA	558071
CD56 Pc5.5	Beckman Coulter, Brea, CA, USA	B49189
MICA/B PE	BioLegend, San Diego, CA, USA	320906
Function		
NKG2D Unconjugated	R&D System, Minneapolis, MN, USA	MAB139
CD226 Unconjugated	BioLegend, San Diego, CA, USA	337102
NKp30 Unconjugated	R&D System, Minneapolis, MN, USA	MAB1849
CD107a PE	BD Biosciences San Diego, CA, USA	555801
IFN-γ Alexa Fluor®647	BD Biosciences San Diego, CA, USA	557729
7-Aminoactinomycin D (7-AAD)	BD Biosciences San Diego, CA, USA	559925

Supplementary Materials and Methods

Isolation of peripheral blood mononuclear cells and tissue-infiltrating lymphocytes

Peripheral blood mononuclear cells (PBMC) were isolated by Lympholyte (Cedarlane, Burlington, Canada) density gradient (1.0770) centrifugation following the manufacture's instruction. Briefly, whole blood was diluted with an equal volume of phosphate-buffered saline (PBS, Sigma-Aldrich, St. Louis, MO, USA) and layered over Lympholyte. After centrifugation at 500 × g for 30 min at room temperature without the brake applied, the PBMC interface was carefully removed by pipetting and washed with PBS with 2% fetal calf serum (FCS, HyClone, GE Healthcare, South Logan, Utah, USA) by centrifugation at 400 × g for 10 min. PBMC were counted by excluding non-viable cells were identified by staining with trypan blue. PBMC were cryopreserved in liquid nitrogen in FCS containing 10 % dimethyl sulfoxide (DMSO; Sigma-Aldrich, St. Louis, MO, USA) and stored until required for downstream analyses.

Tissue samples were treated by mechanical and enzymatic dissociation with the human Tumor Dissociation Kit and by gentleMACS Dissociator (both from Miltenyi Biotec, Bergisch Gladbach, Germany), according to the manufacturer's instructions. The cell suspension was filtered in a 70μm cell strainer (Miltenyi Biotec) and centrifuged twice at 50 × g for 2 min. The supernatant containing lymphocytes was processed for flow cytometry or cryopreserved in liquid nitrogen in FCS containing 10 % DMSO and stored until required for downstream analyses .The tumor cells were plated in culture as described below.

Cell lines and primary HCC cell cultures

Cell cultures were maintained at 37°C with 5% CO2 in a humidified atmosphere. Daudi cells were cultured in RPMI-1640 supplemented with 10% of FCS, 1% antibiotic antimycotic solution, 1% L-glutamine and 1% Sodium Pyruvate (Sigma-Aldrich). The Huh 7.5 cell line (kindly provided by T. Wakita of National Institute of Infectious Diseases, Tokyo, Japan) was maintained in complete Dulbecco's Modified Eagle Medium (DMEM, Thermo Fisher Scientific, Waltham, MA, USA) supplemented with 10% FCS, 10 mM HEPES (Sigma-Aldrich), 1% antibiotic antimycotic solution and 1% L-glutamine. Murine P815 cells were cultured in DMEM supplemented with 10% FCS and 1% antibiotic antimycotic solution.

To establish *in vitro* primary HCC cell cultures, the cell pellet obtained after dissociation of resected tumor tissue was plated in tissue culture flasks (Corning, NY, USA) with DMEM

supplemented with 10% FCS, 1% antibiotic antimycotic solution and 1% non-essential amino acids (Thermo Fisher Scientific) and used after low passages.

Expression of MICA/B was evaluated by labeling Huh 7.5 or HCC cells with anti-MICA/B PE-conjugated (BioLegend, San Diego, CA, USA) or with control IgG isotype and examined by flow cytometry.

Functional assays

Redirecting assay, also named reverse antibody-dependent cellular cytotoxicity (rADCC) assay, was performed after cross-linking of activating receptors and FcγR+ P815 murine cell line. PBMC, TIL and LIL were incubated overnight with or without IL-15 (20 ng/ml, PeproTech EC, London, UK) and subsequently washed and incubated for 4 hours at 37°C with FcγR+ P815 murine target cells (E:T=1:1) in the presence of anti-NKG2D, -CD226 or -NKp30 specific mAbs (BioLegend and R&D System, Minneapolis, MN), anti-CD107a PE and the Protein Transport Inhibitor GolgiStop (both from BD Biosciences, San Diego, CA, USA). After washing, lymphocytes were stained for surface NK cell markers using anti-CD3 BV421™ (BD Biosciences) and anti-CD56 Pc5.5 (Beckman Coulter, Brea, CA, USA). Cells were fixed with BD Cytofix/Cytoperm and permeabilized with the BD Perm/Wash buffer (BD Biosciences) according to the manufacturer's instructions, in the presence of anti-IFNγ Alexa Fluor®647 (BD Biosciences). LIVE/DEAD® Fixable Near-IR Dead Cell Stain Kit was used to determine cell viability. Data analysis was performed with the Kaluza 2.1 software (Beckman Coulter). Antibody-dependent cellular cytotoxicity (ADCC) was evaluated in several experimental conditions. When the Huh 7.5 or primary HCC cells were used as targets, $5x10^5$ cells/well were seeded in 24-well plate in appropriate culture conditions with anti-CD107a PE and co-cultured for 4 hours with effector cells (E:T=5:1), in the presence of 10 μg/ml control isotype (IgG) or human anti-MICA/B mAb (kindly provided by Innate Pharma, Marseille, France). The effectors were then harvested, washed and stained with anti-CD3 BV421™ and anti-CD56 Pc5.5. To evaluate target cell death, primary HCC cells were recovered and labeled with 7-Aminoactinomycin D (7-AAD, BD Biosciences), according to the manufacturer's instructions. When Daudi cells were used as target, the ADCC assay was performed in 96-round-bottom plates by co-culturing effector and target cells (E:T=1:1) for 4 hours at 37°C with anti-CD107a-PE antibody, with or without an anti-CD20 antibody (rituximab, 10 μg/ml). At the end of culture, cells were harvested and labeled with LIVE/DEAD® Fixable Near-IR Dead Cell Stain Kit, CD45 BV786™ (BD Biosciences), CD3 FITC (BD Biosciences) and CD56 Pc5.5.

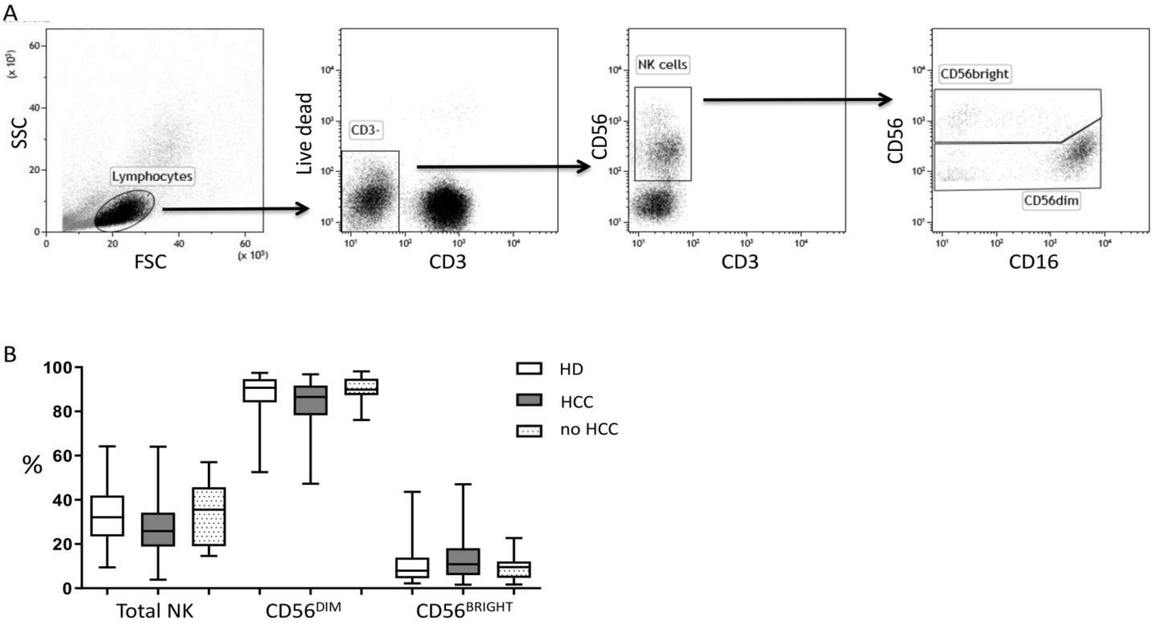

Figure S. Gating strategy in a representative HCC patient **(A)**. Frequencies of total circulating NK and of CD56^{dim} and CD56^{bright} subpopulations in healthy donors (HD, n=60) and in HCC (HCC, n=43) or cirrhotic non-HCC patients (no HCC, n=10) (**B**). Middle bars represent median values, box plots are 25% and 75% percentiles, whiskers are minimum and maximum values. The Mann-Whitney U test was used to compare data.

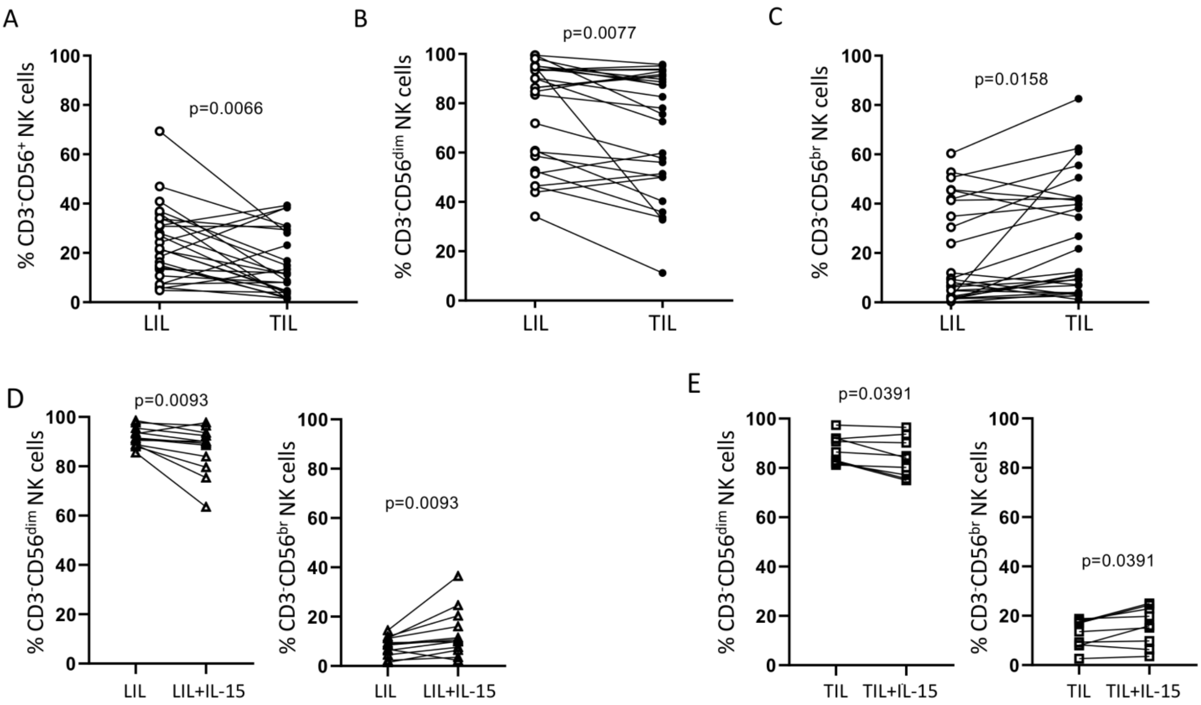

Figure S2. NK cell subset distribution in LIL and TIL. The frequency of total NK cells (**A**) and the distribution of CD56dim (**B**) and CD56bright (**C**) cells were evaluated in LIL and matched TIL (n=26) of resected HCC patients. Proportions of CD56dim and CD56bright NK cells in LIL (D, n=12) and TIL (E, n=9) with or without IL-15 stimulation. The Wilcoxon matched-pairs signed rank test were used to analyze data.

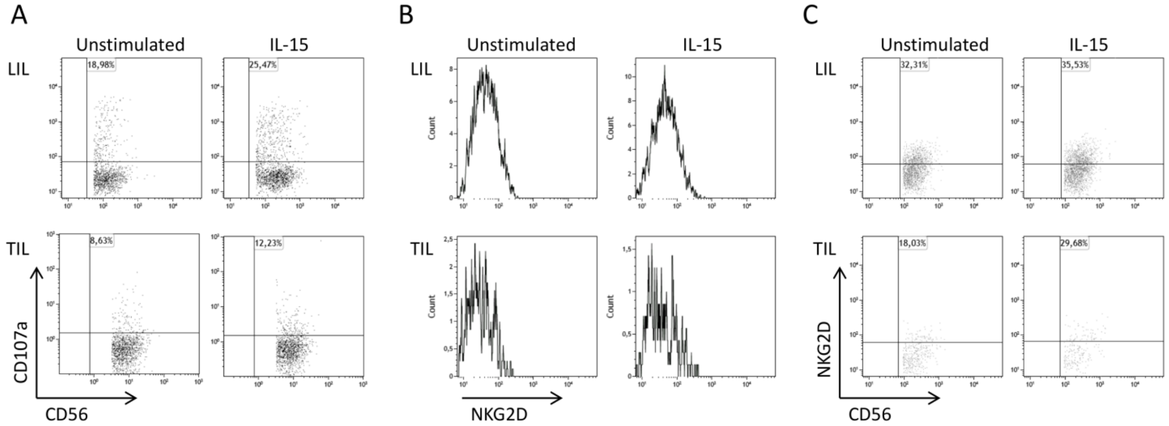

Figure S3. NKG2D-mediated degranulation and NKG2D expression in NK-LIL and NK-TIL. A: Representative dot plots showing NKG2D-mediated cell degranulation of unstimulated or IL-15 stimulated LIL and TIL. **B, C:** Representative histograms and dot plots showing NKG2D expression and frequency in NK-LIL and NK-TIL with or without IL-15 stimulation.

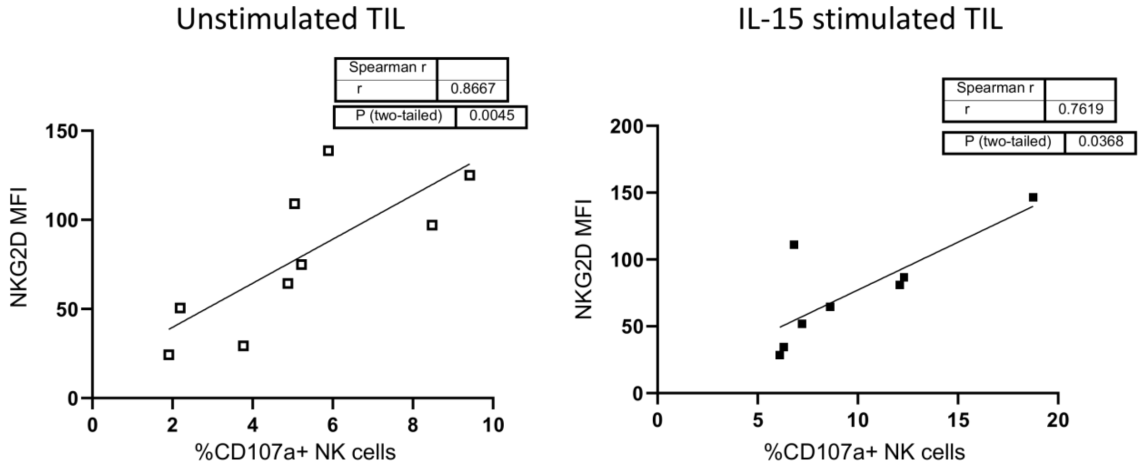

Figure S4. Correlation between NKG2D expression and NK-TIL cell degranulation. Expression of NKG2D (Mean Fluorescence Intensity, MFI) and NK cell cytotoxic activity (%CD107a+ NK cells) of unstimulated or IL-15 stimulated TIL used in rADCC assays were analyzed by non parametric Spearman test.

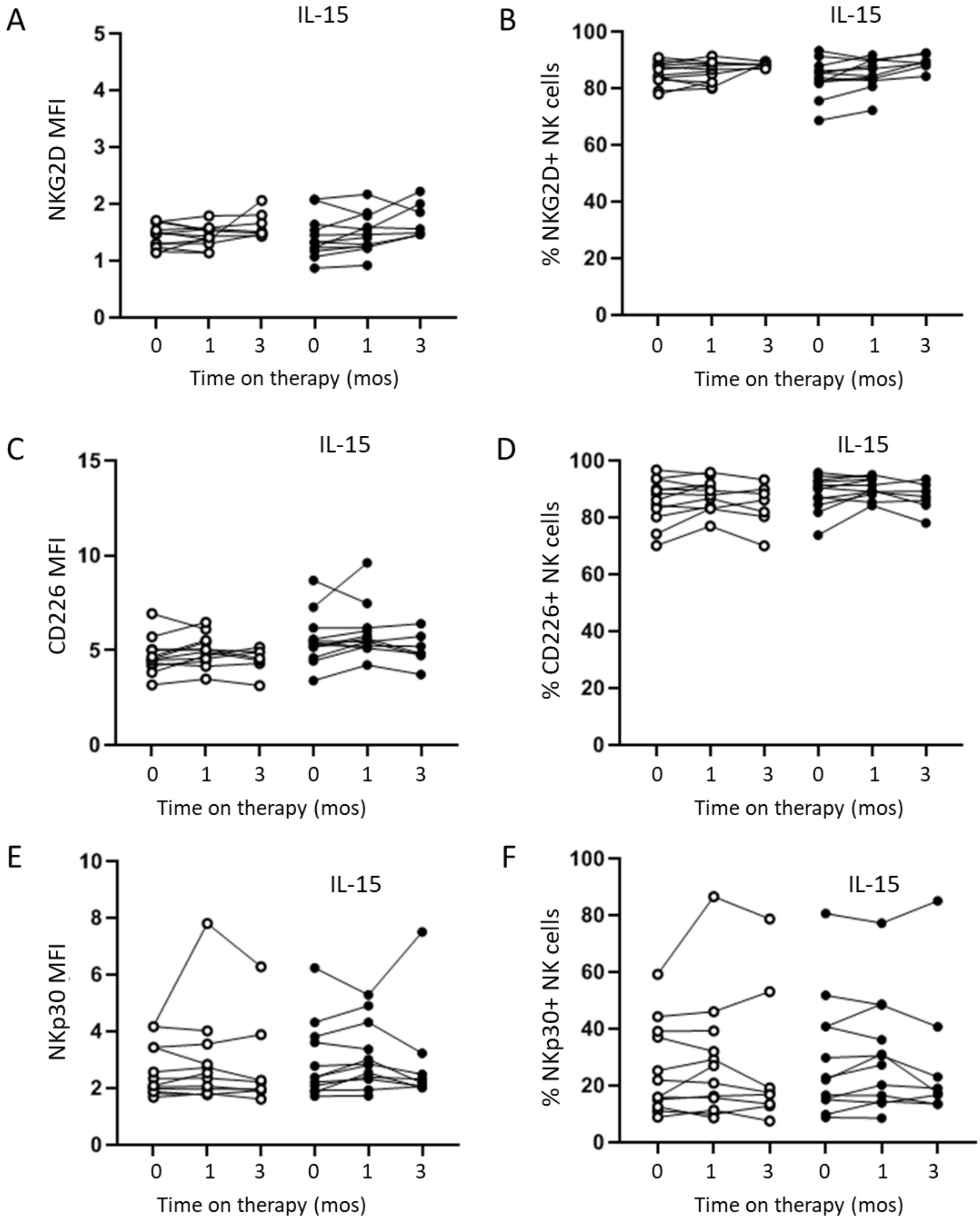

Figure S5. Expression of activating receptors on NK cells of HCC patients treated with sorafenib. Expression (Mean Fluorescence Intensity, MFI) of NKG2D, CD226 and NKp30 (**A, C, E**) and frequencies of NKG2D+, CD226+ or NKp30+ NK cells (**B, D, F**) before or after IL-15 stimulation (IL-15) in HCC patients at baseline (0) and 1 and 3 months after starting sorafenib treatment.

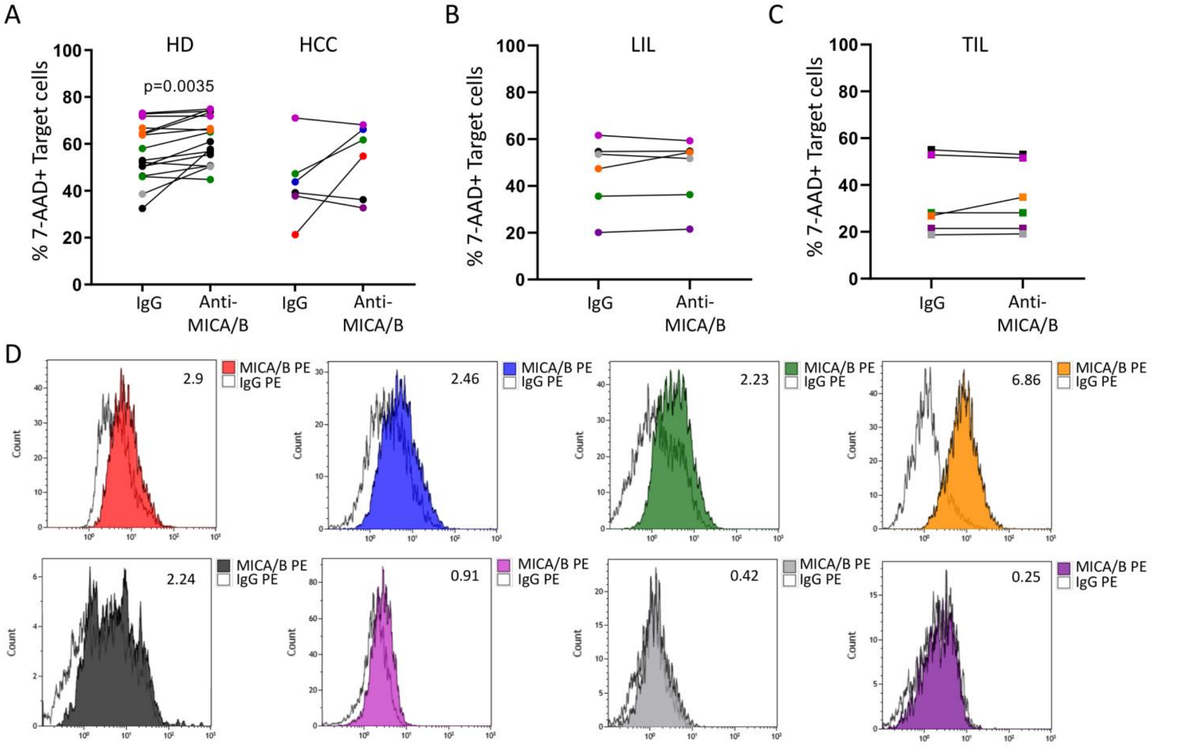

Figure S6. Anti-MICA/B-mediated ADCC. **A**: ADCC toward allogeneic or autologous primary HCC cells by IL-15 stimulated effector PBMC from HD (n=16) and HCC (n=6), respectively. ADCC was evaluated in the presence of control isotype (IgG) or anti-MICA/B mAb (anti-MICA/B) and expressed as percentage of dead target cells (% of 7-AAD+ target cells). ADCC activity of IL-15 stimulated LIL (**B**, n=6) and TIL (**C**, n=6) was measured toward autologous HCC cells as targets, under identical experimental conditions. **D**: Expression of MICA/B in primary HCC cells used in ADCC assays. Colors indicate the corresponding value showed in panels A, B and C. The numbers reported in the histograms represent the differences in MFI between anti-MICA/B and IgG isotype control. The Wilcoxon matched-pairs signed rank test was used to analyze data.

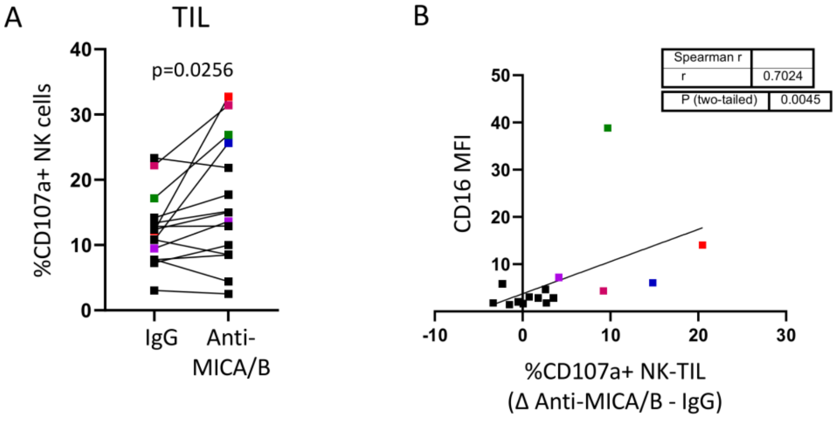

Figure S7. Correlation between CD16 expression and NK-TIL degranulation. A: Degranulation (% CD107a+ NK cells) of NK-TIL in the presence of a control isotype (IgG) or an anti-MICA/B mAb (anti-MICA/B). **B:** Correlation between CD16 expression (Mean Fluorescence Intensity, MFI) on NK-TIL and the difference between degranulation with anti-MICA/B mAb and IgG isotype control (Δ Anti-MICA/B - IgG).

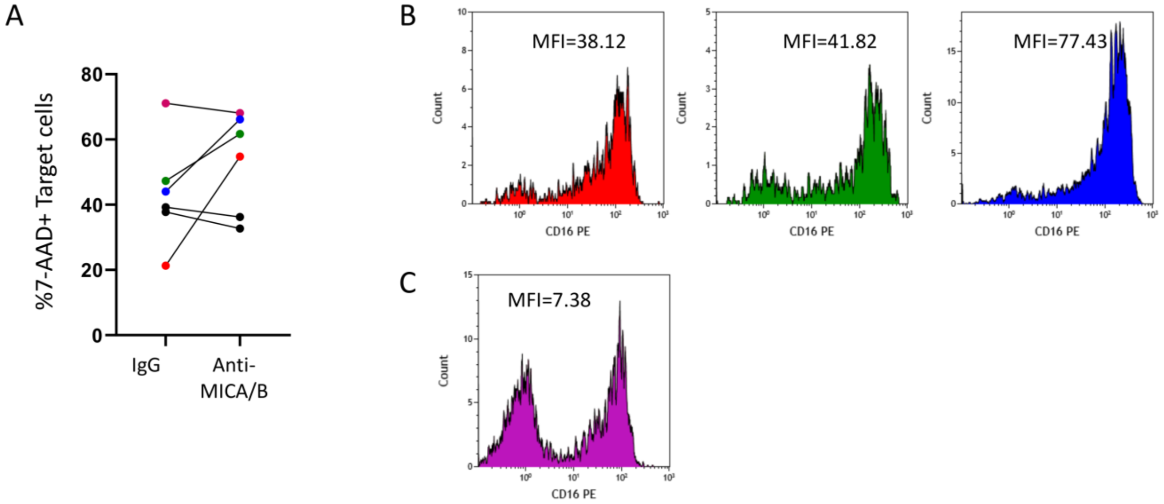

Figure S8. CD16 expression on PB NK and their killing activity A: Primary HCC target cells lysis (% 7-AAD+ Target cells) by autologous PB NK of HCC patients. Representative histograms showing the CD16 MFI of responding (**B**) or non responding (**C**) PB NK cells used in ADCC assays.
